# Asymmetric Synthesis of 4,1-Benzoxazepine-2,5-Diones — Effect of the Halogen of (2*S*)-α-Haloacids

**DOI:** 10.3390/molecules19010139

**Published:** 2013-12-23

**Authors:** Syeda Laila Rubab, Bushra Nisar, Abdul Rauf Raza, Nisar Ullah, Muhammad Nawaz Tahir

**Affiliations:** 1 Ibn-e-Sina Block, Department of Chemistry, University of Sargodha, Sargodha 40100, Pakistan; E-Mails: xahrachemist@gmail.com (S.L.R.); b.nisacapri@gmail.com (B.N.); 2 Chemistry Department, King Fahd University of Petroleum and Minerals, Dhahran 31261, Saudi Arabia; 3 Department of Physics, University of Sargodha, Sargodha 40100, Pakistan; E-Mail: dmntahir_uos@yahoo.com

**Keywords:** (3*R*)-3-alkyl-4,1-benzoxazepine-2,5-diones, asymmetric synthesis, α-chloroacids, α-bromoacids, anthranilic acid, chiral pool methodology

## Abstract

Novel chiral 4,1-benzoxazepine-2,5-diones have been unusually synthesized in a single step by exploiting the chiral pool methodology. Substituted anthranilic acids afford *N*-acylanthranilic acids and (3*R*)-3-alkyl-4,1-benzoxazepines-2,5-dione upon coupling with α-chloroacids or α-bromoacids, respectively.

## 1. Introduction

The benzoxazepines belongs to the heterocycles class of compounds, which are obligatory components of biologically important molecules such as nucleic acids, hormones and therapeutic drugs. The benzoxazepine scaffolds are very versatile and of therapeutic use in many important fields. They have acquired tremendous importance in recent years owing to their wide applications in the medicinal and pharmaceutical industry. For example, benzoxazepines have shown anti-tumor [1,2], anti-HIV [3], and tranquilizing activities [4], among a long list of other effects. Most of the conventional synthesis methods reported in literature produce *racemic*/achiral syntheses of 4,1-benzoxazpine. Leptit* et al.* reported the synthesis of 4,1-benzoxazepine by *N*-alkylation of 2-amino benzhydrol, followed by cyclization in the presence of ethanolic Na solution to yield 5-phenyl-1,3,5-trihydro-4,1-benzoxazepine-2-ones [5]. Bergman* et al.*, reported *N*-alkylation of *N*-methylanthranilic acid with an α-chloroacid followed by intramolecular cyclization to afford 4,1-benzoxazepine-3,5-dione [6]. Yar *et al.*, reported a single step synthesis of 4,1-benzoxazepine in which *N*-tosyl-1,3-aminoalcohols were treated with bromoethylsulfonium salts, via vinyl sulfonium salt formation, which upon intramolecular cyclization afforded 4,1-benzoxazepines [7]. Because of the variety of their biological activities, these are heterocycles of intense chemical and biological significance.

Asymmetric synthesis is acquiring greater significance in pharmaceutical industry because of the wider application of enantiopure drugs. Mostly medicines used are *racemic modifications* of two enantiomers and side-effect of these medicines is being found due to presence of the vestigial enantiomers [8,9]. Asymmetric synthesis of thus heterocycles attracting greater attention in synthetic chemistry. The accessibility of drug for a community depends on the cost as well. A more efficient drug with high purchase value may not be accessible for all economical levels. This can be avoided by using inexpensive starting materials, especially, those from natural sources. Our methodology employs the chiral pool strategy that involves the use of absolutely enantiopure starting materials, which can be obtained easily from natural resources and tailors a/several chiral centre(s) in a target molecule with up to 100% stereoselectivity. The natural amino acids are inexpensive and readily available chiral starting materials. Many strategies reported in the literature are inspired by chiral pool methodology which makes use of naturally occurring chiral amino acids [10,11]. Our previous work also involved chiral pool strategy which employs inexpensive (*S*)-amino acids as starting materials to afford (3*R*)-4,1-benzoxazepines in high *ee* (up to 81%) [12].

## 2. Results and Discussion

**Scheme 1:**
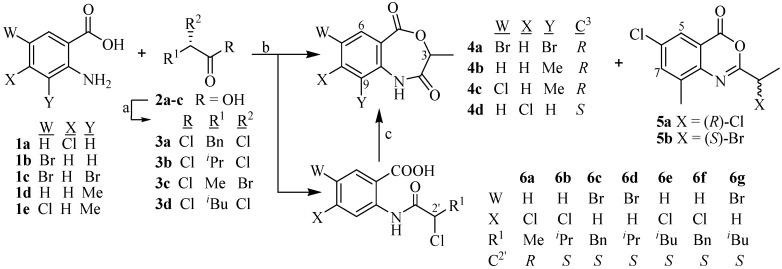
Synthesis of *N*-acylanthranilic acids **6a**–**g** and benzoxazepines **4a**–**d**.

This strategy involved chiral pool methodology in which chiral substrates are coupled with achiral anthranilic acids to afford chiral 4,1-benzoxazepine-2,5-diones. We planned to synthesize 4,1-benzoxazepines in two steps, which involve the coupling of α-haloacids with various anthranilic acids followed by intramolecular cyclization to afford the corresponding 4,1-benzoxazepine-2,5-diones. For this purpose (−)-(*S*)-2-chloroacids or (−)-(*S*)-2-bromoacids **3a**–**c** were prepared in high *ee* (95%–98%) *via* diazotization of naturally occurring (+)-(*S*)-amino acids [13]. The coupling of α-chloroacids **3a**–**b** with **1a**–**e** afforded *N*-acylanthranilic acid as expected, but the use of α-bromoacid **3c** resulted in the formation of seven member ring compounds **4a**–**c** in most cases (Scheme 1 and Table 1).

The reaction of anthranilic acid **1a**–**e** with α-chloroacids **3a**–**b** and **3d** affords (3*S*)-*N*-acylanthranilic acids **6b**–**g**. When the reaction mixture was poured into ice chilled H_2_O the compounds **6a**–**c** precipitated out as white solids that were purified by crystallization from EtOAc, whilst **6d**–**g** were purified by column chromatography. However, under such conditions the coupling of **1a** and **1c**–**e** with (*S*)-2-bromopropanoic acid **3c** affords either (3*R*)-4,1-benzoxazepines **4a**–**c** as a major product in most cases or (3*S*)-*N*-acylanthranilic acid **6a** after transhalogenation. The Br atom is a good leaving group and it is replaced by a Cl ion (transhalogenation), because chlorine ion is present in the reaction mixture resulting in the formation of (*R*)-2-chloropropanoic acid which upon coupling with anthranilic acids gave a mixture of both Cl- and Br-substituted products.

*Reagents and Conditions*: (**a**) SOCl_2_ (1.5 eq), DMF (1 drop); (**b**) dropwise addition of **3** to **1**, DMF, 0 °C; (**c**) K_2_CO_3_, DMF, 80 °C (3 h).

**Table 1:** The %yield and specific rotations of **4a**–**d**, **5** and **6a**–**g**.

	4a	4b	4c	4d	5 ^§^	6a	6b	6c	6d	6e	6f	6g
% Yield	50	78	66	57	32	67	86	70	71	68	67	46
c *	0.5	0.2	0.2	0.2	0.3	0.5	0.6	1.0	1.0	1.0	1.0	0.2
[α]_D_ (°C)	+12.0 (30)	+80.0 (30)	+67.9 (30)	+54.0 (30)	–19.0 (23)	+80.0 (30)	+16.9 (30)	+17.0 (30)	+32.0 (30)	+16.2 (25)	+35.2 (25)	+23.3 (26)

* MeOH, taken in g/100 mL unit and measured in a cell of 1 dm length; ^§^ mixture of both **5a** and **5b** (82:18).

The coupling of **1c**–**e** with **3c** affords the unusual (3*R*)-4,1-benzoxazepines **4a**–**c** in the majority of the cases. The Br is replaced by either the O of the COOH group in *N*-acylanthranilic acid or with the Cl ion to afford 4,1-benzoxazepine directly or the Cl-substituted *N*-acylanthranilic acid, respectively. The coupling of **1a** with **3c** afforded the Cl-substituted *N*-acylanthranilic acid **6a** exclusively. In this case, the Br is replaced by the Cl ion during the acid halide formation of (*S*)-2-bromopropanoic acid **3c** with SOCl_2_.

**Scheme 2:**
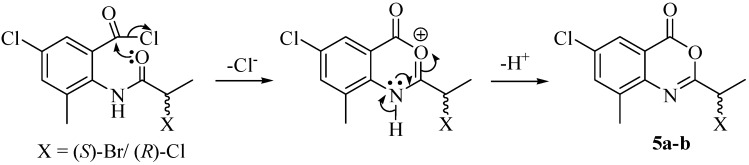
Mechanism showing the formation of 6-chloro-2-(1'-haloethyl-8-methyl-3,1-benzoxazine-4-one **5a**–**b**.

The formation of Cl-substituted *N*-acylanthranilic acid in **6a** was confirmed by the appearance of the molecular ion observed in LR EIMS; the [M]^+^ appeared at 261, 263 and 265 amu in a 9:6:1 ratio that proves the transhalogenation (presence of two Cl) has ocurred. It is observed that a slight excess of SOCl_2_ (2 eq) in the acid halide formation step affords benzoxazinones **5a/5b** as a side-product along with 4,1-benzoxazepine **4c** as major product. It is proposed that *N*-acylanthranilic acid reacts with SOCl_2_ to form the acid halide which undergoes cyclization to the six member benzoxazinones **5a/5b** (Scheme 2).

**Figure 1:**
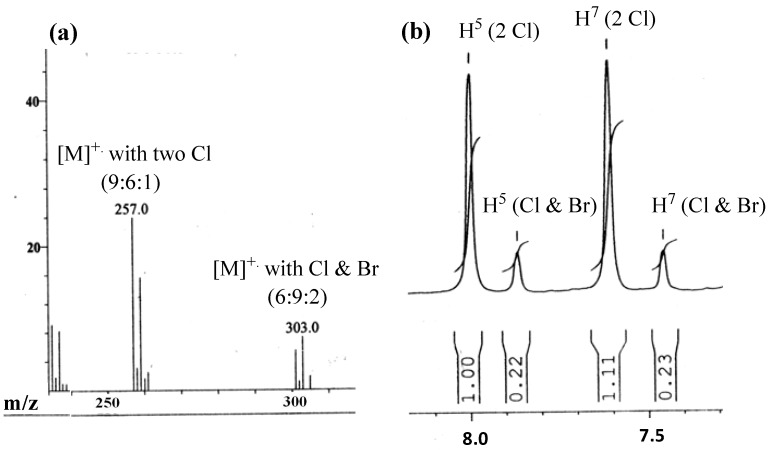
A part of (**a**) LR EIMS showing the predominance of **5a** molecular ions; (**b**) the ^1^H-NMR elaborating the level of predominance of Cl-substituted benzoxazinone **5a** over Br-substituted benzoxazinone **5b**.

The benzoxazinone is also a mixture of two products **5a/5b** (both Cl- and Br-substituted) in which mainly the Cl-substituted benzoxazinone **5a** dominates. Transhalogenation was confirmed by the presence of both Cl- and Br-substituted molecular ion signals in 9:6:1 and 6:9:2 respectively, observed in LR EIMS (Figure 1a).

The LR EIMS revealed molecular ion signals at 301, 303, 305 and 257, 259, 261 amu which confirm the presence of both Br and Cl atoms at C^2^ respectively. It shows that the Cl-substituted product **5a** dominates over the Br-substituted product **5b** since the signals of the former radical cation show more abundance in the LR EIMS. Each aromatic proton shows a pair of signals of unequal size in ^1^H NMR (Figure 1b); the integration of each signal pair revealed the level of predominancy of the Cl-substituted compound **5a** (82%) over Br substituted benzoxazinone **5b** (18%).

**Figure 2:**
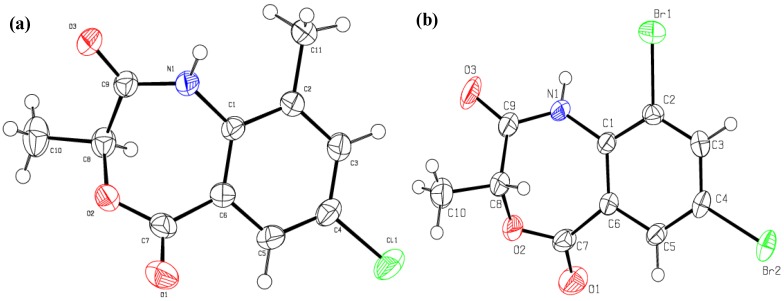
The ORTEP diagram of; (**a**) **4c**; (**b**) **4a**.

In the case of **6a**–**g** no cyclized product was formed; probably the ease of C-Br bond dissociation is the reason for such behavior (direct cyclization). The Br is a better leaving group than Cl and for cyclization of the Cl substituted *N*-acylanthranilic acids **6a**–**g**, base (K_2_CO_3_) catalysis is required to get the cyclized 4,1-benzoxazepines [12]. The formation of 4,1-benzoxazepine in a single step was confirmed by single crystal XRD (Figure 2), which indicates the disappearance of C-Br and the formation of a new C-O bond (1.455 and 1.448 Å in **4c** and **4a** respectively) [14].

The base mediated the intramolecular cyclization of the *N*-acylated anthranilic acid **6a**, obtained by the coupling of anthranilic acid **1a** with acid chlorides **3c**, to afford the 4,1-benzoxazepine-2,5-dione **4d** that was purified by column chromatography. The ^1^H-NMR spectra of the 4,1-benzoxazepine **4d** showed no prominent changes as compared to the *N*-acylated anthranilic acid precursor **6a**. A small shift is observed for the proton present at the chiral centre, which appeared slightly downfield (δ = 4.79 ppm) as compared to corresponding precursor acid **6a** (δ = 4.45 ppm) due to the electron withdrawing inductive effect of O.

## 3. Experimental

### General Information

The pre-coated silica gel (0.25 mm thick layer over Al sheet, Merck, Darmstadt, Germany) TLC plates were used to monitor the reactions. Glass column packed silica gel (0.6–0.2 mm, 60 Å mesh size, Merck) were used for purification. The ^1^H-NMR and ^13^C-NMR were recorded in the designated solvents on a Bruker AVANCE DPX (300, 400, 500 or 600 MHz) spectrometer (Bruker, Billarica, MA, USA) using TMS as internal standard. The optical rotation was measured on an Atago (AP-300) polarimeter (Atago, Tokyo, Japan). The HR ESI was recorded on a Q-TOF Ultima API instrument (Micromass, Waters, Milford, MA, USA) at the Biomedical Mass Spectrometry Facility (BMSF), UNSW, Sydney (Australia). The single crystal X-Ray data were recorded on a Bruker Kappa APEX 11 CCD diffractometer. The IR and UV/Vis spectra were recorded on a Prestige 21 FTIR spectrometer (Shimadzu, Tokyo, Japan) and a Thermo Spectronic UV-1700 spectrophotometer (Thermo, Waltham, MA, USA), respectively.

Representative procedure for the synthesis of **4a**–**c**, **5a**–**b** and **6a**–**g**: A mixture of (*S*)-2-bromopropanoic acid (5 mmol, 2 eq), SOCl_2_ (7.5 mmol, 2.5 eq) and catalytic amount of DMF (1 drop) was heated at 60 °C for 30 min. The resulting 2-haloacid chlorides **3a**–**d**, without further purification, was slowly added dropwise to a stirred chilled solution of 5-cholo-3-methylanthranilic acid (**1e**) (2.5 mmol, 1 eq) and Et_3_N (2.5 mmol, 1 eq) in DMF (2 mL) at 0 °C. The reaction mixture was stirred overnight at room temperature. An excess of H_2_O was added and extracted with EtOAc (3 × 15 mL). The combined organic layer was dried over anhydrous Na_2_SO_4_, filtered and concentrated under reduced pressure till a brownish liquid was obtained. This crude product was subjected to column chromatography on silica gel that afforded **5** (200 mg, 32%) and/or **4** (390 mg, 66%), both as white solids, by elution with 2% and 5% EtOAc in *n*-hexane.

*(3R)-7,9-Dibromo-3-methyl-4,1-benzoxazepine-2,5-dione* (**4a**): R*_f_*: 0.75 (EtOAc/*n*-hexane 3:7), 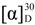 = +12.0 (*c* 0.5, MeOH); MP: 165 °C; ^1^H-NMR (300 MHz, CD_3_OD): δ (ppm) 2.60 (3H, d, *J* = 6.9 Hz, CH_3_), 4.35 (1H, q, *J* = 6.9 Hz, H^3^), 7.70 (1H, d, *J* = 1.5 Hz, H^8^), 7.94 (1H, d, *J* = 1.8 Hz, H^6^); IR (KBr): ύ_max_ (cm^−1^) 1697 (a broad signal of OC=O and NC=O); UV-Vis (MeOH): λ_max_ 304 nm (log ε = 3.21670 L cm^−1^ M^−1^); LR EIMS: *m/z* in amu (% abundance) 351, 349, 347 (6, 12, 6 in 1:2:1 ratio) [M]^+•^, 279, 277 and 275 (39, 77 and 40 in 1:2:1 ratio) [M-C_3_H_4_O_2_, A]^+•^, 251, 249 and 247 (8, 17 and 9 in 1:2:1 ratio) [A-CO]^+•^, ESI MS (*m/z*) for C_10_H_7_Br_2_NO_3_: 373.8649, 371.8669 and 369.8690 found for 373.8645 [M+4+Na], 371.8665 [M+2+Na] and 369.8686 [M+Na] in 1:2:1.

*(3R)-3,9-Dimethyl-4,1-benzoxazepine-2,5-dione* (**4b**): R*_f_*: 0.65 (EtOAc/*n*-hexane 3:7), 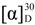 = +80.0 (*c* 0.2, MeOH); MP: 190 °C; ^1^H-NMR (300 MHz, CDCl_3_): δ (ppm) 1.83 (3H, d, *J* = 6.9 Hz, CH_3_), 2.27 (3H, s, Ar-CH_3_), 4.55 (1H, q, *J* = 6.9 Hz, H^3^), 7.24 (1H, t, *J* = 7.5 Hz, H^7^), 7.49 (1H, d, *J* = 7.5 Hz, H^8^), 7.90 (1H, d, *J* = 7.8 Hz, H^6^), 9.56 (1H, s, NH); ^13^C-NMR (75 MHz, CDCl_3_): δ (ppm) 18.8 (Ar-CH_3_), 22.8 (C^1^^'^), 55.9 (C^3^), 126.2 (C^9^), 126.3 (C^7^), 129.3 (C^8^), 129.4 (C^5a^), 136.5 (C^6^), 136.6 (C^9a^), 168.0, 170.6 (C^2^ and C^5^); IR (KBr): ύ_max_ (cm^−1^) 1697 (a broad signal of OC=O and NC=O); UV-Vis (MeOH): λ_max_ 294 nm (log ε = 3.32135 L cm^−1^ M^−1^); LR EIMS: *m/z* in amu (% abundance) 205 (72) [M]^+•^, 133 (100) [M-C_3_H_4_O_2_, A]^+•^, 105 (100) [A-CO]^+•^, ESI MS (*m/z*) for C_11_H_11_NO_3_: 228.0636 found for 228.0631 [M+Na].

*(3R)-7-Chloro-3,9-dimethyl-4,1-benzoxazepine-2,5-dione* (**4c**): R*_f_*: 0.61 (EtOAc/*n*-hexane 3:7); 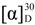 = +67.9 (*c* 0.2, EtOAc); MP: 187 °C; ^1^H-NMR (300 MHz, CD_3_OD): δ (ppm) 1.73 (3H, d, *J* = 6.9 Hz, H^1^^'^), 2.26 (3H, s, Ar-CH_3_), 4.67 (1H, q, *J* = 6.9 Hz, H^3^), 7.50 (1H, broad s, H^8^), 7.76 (1H, d, *J* = 2.4 Hz, H^6^); ^13^C-NMR (75 MHz, CD_3_OD): δ (ppm) 18.2 (Ar-CH_3_), 22.1 (C^1^^'^), 55.7 (C^3^), 129.4 (C^8^), 129.8 (C^5a^), 135.0 (C^6^), 135.6 (C^7^), 168.0, 170.6 (C^2^ and C^5^); IR (KBr): ύ_max_ (cm^−1^) 3362 (N-H), 1693 (a broad signal of OC=O and NC=O); UV-Vis (MeOH): λ_max_ 306 nm (log ε = 3.25701 L cm^−1^ M^−1^); LR EIMS: *m/z* in amu (% abundance) 241, 239 (13, 37 in 1:3 ratio) [M]^+•^, 169, 167 (29, 100 in 1:3 ratio) [M-(3-methyloxirane-2-one), A]^+•^, 141, 139 (31, 90 in 1:3 ratio) [A-CO]^+•^; ESI MS (*m/z*) for C_11_H_10_ClNO_3_: 264.0217 and 262.0246 found for 264.0214 [M+2+Na] and 262.0242 [M+Na] in 1:3 ratio.

*(1'R)-6-Chloro-2-(1'-chloroethyl)-8-methyl-3,1-benzoxazine-4-one* (**5a**): R*_f_*: 0.87 (EtOAc/*n*-hexane 3:7); 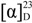 = −19.0 (*c* 0.3, EtOAc); MP: 129 °C; ^1^H-NMR (300 MHz, CDCl_3_): δ (ppm) 2.04 (3H, d, *J* = 6.9 Hz, H^2^^'^), 2.53 (3H, s, Ar-CH_3_), 4.82 (1H, q, *J* = 6.9 Hz, H^1^^'^), 7.61 (1H, broad s, H^7^), 8.00 (1H, broad s, H^5^), IR (KBr): ύ_max_ (cm^−1^) 1764 (lactonic OC=O), 1528 (C=N); UV-Vis (MeOH): λ_max_ 326 nm (log ε = 3.96534 L cm^−^^1^ M^−^^1^); LR EIMS: *m/z* in amu (% abundance) 261, 259 and 257 (2.5, 16 and 24 in 1:6:9 ratio) [M with 2 Cl]^+•^, 224, 222 (22, 63 in 1:3 ratio) [M-^•^Cl]^+^, 196, 194 (31, 100 in 1:3 ratio) [M-H_3_CC**∙**(H)Cl]^+^; ESI MS (*m/z*) for C_11_H_9_Cl_2_NO_2_: 283.9849, 281.9878 and 279.9908 found for 283.9846 [M+4+Na], 281.9875 [M+2+Na] and 279.9905 [M+Na] in 1:6:9 ratio.

*(1'S)-2-(1'-Bromoethyl)-6-chloro-8-methyl-3,1-benzoxazine-4-one* (**5b**): R*_f_*: 0.87 (EtOAc/*n*-hexane 3:7); 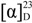 = −19.0 (*c* 0.3, EtOAc); MP: 129 °C; ^1^H-NMR (300 MHz, CDCl_3_): δ (ppm) 1.95 (3H, d, *J* = 6.6 Hz, H^2^^'^), 2.24 (3H, s, Ar-CH_3_), 4.51 (1H, q, *J* = 6.9 Hz, H^1^^'^), 7.45 (1H, broad s, H^7^), 7.86 (1H, broad s, H^5^), IR (KBr): ύ_max_ (cm^−1^) 1761 (lactonic OC=O), 1528 (C=N); UV-Vis (MeOH): λ_max_ 326 nm (log ε = 3.96534 L cm^−^^1^ M^−^^1^); LR EIMS: *m/z* in amu (% abundance) 305, 303 and 301 (1.9, 7.4 and 5.5 in 2:9:6 ratio) [M with Br and Cl]^+•^, 224, 222 (22, 63 in 1:3) [M-^•^Br]^+^, 196, 194 (31, 100 in 1:3 ratio) [M-H_3_CC^•^(H)Br]^+^; ESI MS (*m/z*) for C_11_H_9_BrClNO_2_: 327.9352, 325.9373 and 323.9402 found for 327.9350 [M+4+Na], 325.9370 [M+2+Na] and 323.9400 [M+Na] in 2:9:6 ratio.

*(2'R)-4-Chloro-2-(2'-chloropropanamido)benzoic acid* (**6a**): The product (0.39 g, 67%) was precipitated out when reaction mixture was poured into ice chilled H_2_O. R*_f_*: 0.56 (EtOAc/*n*-hexane 3:7); 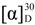 = +80.0 (*c* 0.5, EtOAc); MP: 187 °C; ^1^H-NMR (400 MHz, CDCl_3_): δ (ppm) 1.82 (3H, d, *J* = 7.2 Hz, H^3^^'^), 4.54 (1H, q, *J* = 7.2 Hz, H^2^^'^), 7.15 (1H, dd, *J* = 8.4, 1.6 Hz, H^5^), 8.07 (1H, d, *J* = 8.8 Hz, H^6^), 8.82 (1H, d, *J* = 1.5 Hz, H^3^), 11.7 (1H, s, NH); IR (KBr): ύ_max_ (cm^−1^) 1678 (OC=O), 1583 (NC=O); UV-Vis (MeOH): λ_max_ 306 nm (log ε = 3.950121 L cm^−1^ M^−1^); LR EIMS: *m/z* in amu (% abundance) 265, 263 and 261 (2, 11 and 21 in 1:6:9 ratio) [M]^+•^, 200, 198 (15, 45 in 1:3 ratio) [M-H_3_CC^•^(H)Cl, A]^+^, 182, 180 (45, 100) [A-H_2_O]^+^.

*(2'S)-4-Chloro-2-(2'-chloro-3'-methylbutanamido)benzoic acid* (**6b**): R*_f_*: 0.45 (EtOAc/*n*-hexane 3:7), 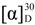 = +16.9 (*c* 0.6, MeOH); MP: 178 °C; ^1^H-NMR (300 MHz, CDCl_3_): 1.00 (3H, d, *J* = 6.3 Hz, CH_3_), 1.09 (3H, d, *J* = 6.6 Hz, H^4^**^'^**), 2.55 (1H, m, H^3^**^'^**), 4.35 (1H, d, 4.5 Hz, H^2^**^'^**), 7.13 (1H, dd, *J* = 8.7, 1.5 Hz, H^5^), 8.06 (1H, d, *J* = 8.6 Hz, H^6^), 8.83 (1H, d, *J* = 1.5 Hz, H^3^), 11.78 (1H, s, NH); IR (KBr): ύ_max_ (cm^−1^) 3500 (O-H), 3383 (N-H), 1666 (OC=O), 1595 (NC=O); UV-Vis (MeOH): λ_max_ 336 nm (log ε = 3.01872 L cm^−1^ M^−1^). ESI MS (*m/z*) for C_12_H_13_Cl_2_NO_3_: 316.0111, 314.0140 and 312.0170 found for 316.0108 [M+4+Na], 314.0136 [M+2+Na] and 312.0167 [M+Na] in 1:6:9 ratio.

*(2'S)-5-Bromo-2-(2'-chloro-3'-phenylpropanamido)benzoic acid* (**6c**): R*_f_*: 0.14 (EtOAc/*n*-hexane 3:7), 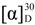 = +17.0 (*c* 1.0, MeOH); MP: 110 °C; ^1^H-NMR (500 MHz, CD_3_OD): 3.21 (1H, dd, *J* = −14.0, 8.0 Hz, H_α_^3^^'^), 3.43 (1H, dd, *J* = −14.0, 8.0 Hz, H_β_^3^^'^), 4.69 (1H, dd, *J* = 8.0, 6.0 Hz, H^2^^'^), 7.17-7.24 (5H, m, Ph), 7.63 (1H, dd, *J* = 9.0, 2.5 Hz, H^4^), 8.11 (1H, d, *J* = 2.5 Hz, H^6^), 8.48 (1H, d, *J* = 9.0, H^3^); ^13^C-NMR (125 MHz, CD_3_OD) δ (ppm): 42.46 (C^3^^'^), 62.29 (C^2^^'^), 116.76 (C^1^^''^), 119.97 (C^1^), 123.08 (C^4^^''^), 128.18 (C^3^), 129.45, 130.54 (C^3^^''^ and C^2^^''^), 134.92 (C^4^), 137.47 (C^5^), 137.71 (C^6^), 140.58 (C^2^), 169.23 (NC=O), 169.57 (OC=O); IR (KBr): ύ_max_ (cm^−1^) 3028 (O-H), 2916 (N-H), 1709 (OC=O), 1531 (NC=O); UV-Vis (MeOH): λ_max_ 317 nm (log ε = 3.56741 L cm^−1^ M^−1^); LR EIMS: *m/z* in amu (% abundance) 385, 383, 381 (1, 5, 3 in 2:9:6 ratio) [M]^+•^, 226, 224 (16, 15 in 1:1 ratio) [M-^•^CH_2_(Cl)Bn and H_2_O, A]^+^, 217, 215 (53, 52 in 1:1) [M-^•^CH_2_(Cl)Bn and CO]^+^, 198, 196 (56, 56) [A-CO]^+^.

*(2'S)-5-Bromo-2-(2'-chloro-3'-methylbutanamido)benzoic acid* (**6d**): R*_f_*: 0.45 (EtOAc/*n*-hexane 3:7), 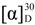 = +32.0 (*c* 1.0, MeOH); MP: 178 °C; ^1^H-NMR (300 MHz, CDCl_3_): 1.00 (3H, d, *J* = 6.3 Hz, CH_3_), 1.09 (3H, d, *J* = 6.6 Hz, H^4^^'^), 2.55 (1H, m, H^3^^'^), 4.35 (1H, d, 4.5 Hz, H^2^^'^), 7.13 (1H, dd, *J* = 8.7, 1.5 Hz, H^4^), 8.06 (1H, d, *J* = 8.6 Hz, H^3^), 8.83 (1H, d, *J* = 1.5 Hz, H^6^), 11.78 (1H, s, NH); IR (KBr): ύ_max_ (cm^−1^) 3500 (O-H), 3383 (N-H), 1666 (OC=O), 1595 (NC=O); UV-Vis (MeOH): λ_max_ 336 nm (log ε = 3.01872 L cm^−1^ M^−1^); ESI MS (*m/z*) for C_12_H_13_BrClNO_3_: 359.9615, 357.9644 and 355.9665 found for 359.9612 [M+4+Na], 357.9642 [M+2+Na] and 355.9663 [M+Na] in 2:9:6 ratio.

*(2'S)-4-Chloro-2-(2'-chloro-3'-methylpentanamido)benzoic acid* (**6e**): R*_f_*: 0.15 (EtOAc/ *n*-hexane 3:7), 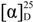 = +16.2 (*c* 1.0, MeOH); MP: 126 °C; ^1^H-NMR (600 MHz, CD_3_OD): 0.96 (3H, d, *J* = 6.6 Hz, CH_3_), 0.98 (3H, d, *J* = 6.6 Hz, H^5^^'^), 1.85–1.98 (3H, m, H^3^^'^ and H^4^^'^), 4.51 (1H, dd, *J* = 9.6, 4.8 Hz, H^2^^'^), 7.18 (1H, dd, *J* = 8.4, 1.8 Hz, H^5^), 8.06 (1H, d, *J* = 8.4 Hz, H^6^), 8.69 (1H, d, *J* = 1.8, H^3^); ^13^C-NMR (125 MHz, CD_3_OD) δ (ppm): 21.56 (Me), 23.06 (C^5^^'^), 26.52 (C^4^^'^), 45.44 (C^3^^'^), 60.48 (C^2^^'^), 116.42 (C^1^), 120.97 (C^5^), 124.53 (C^3^), 133.99 (C^6^), 141.13 and 142.81 (C^2^ and C^4^), 170.39 (NC=O), 170.44 (OC=O); IR (KBr): ύ_max_ (cm^−1^) 3221 (O-H), 3120 (N-H), 1640 (OC=O), 1550 (NC=O); UV-Vis (EtOAc): λ_max_ 307 nm (log ε = 3.44321 L cm^−1^ M^−1^); LR EIMS: *m/z* in amu (% abundance) 307, 305 and 303 (0.1, 1.2 and 2.1 in 1:6:9 ratio) [M]^+•^, 251, 249 and 247 (6, 45 and 77 in 1:6:9 ratio) [M-C_4_H_8_, A]^+•^, 200, 198 (3, 7 in 1:3) [A-^•^CH_2_Cl, B]^+^, 182, 180 (37, 80) [B-H_2_O, C]^+^, 173, 171 (29, 100) [B-CO]^+^, 155, 153 (17, 52) [C-CO]^+^.

*(2'S)-4-Chloro-2-(2'-chloro-3'-phenylpropanamido)benzoic acid* (**6f**): R*_f_*: 0.17 (EtOAc/*n*-hexane 1:1), 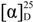 = +35.2 (*c* 1.0, MeOH); MP: 121 °C; ^1^H-NMR (500 MHz, CD_3_OD): 3.23 (1H, dd, *J* = −14.0, 7.5 Hz, H_α_^3^^'^), 3.44 (1H, dd, *J* = −14.0, 6.0 Hz, H_β_^3^^'^), 4.71 (1H, dd, *J* = 7.5, 6.0 Hz, H^2^^'^), 7.15 (1H, dd, *J* = 8.5, 2.0 Hz, H^5^), 7.19-7.26 (5H, m, Ph), 8.01 (1H, d, *J* = 8.5 Hz, H^6^), 8.66 (1H, d, *J* = 2.0, H^3^); ^13^C-NMR (125 MHz, CD_3_OD) δ (ppm): 42.43 (C^3^^'^), 62.24 (C^2^^'^), 116.35 (C^1^^''^), 120.91 (C^5^), 124.55 (C^3^), 128.12 (C^4^^''^), 129.47, 130.57 (C^3^^''^ and C^2^^''^), 133.91 (C^6^), 137.45 (C^1^), 141.04 (C^4^), 142.56 (C^2^), 169.46 (NC=O), 170.18 (OC=O); IR (KBr): ύ_max_ (cm^−1^) 3221 (O-H), 3001 (N-H), 1670 (OC=O), 1543 (NC=O); UV-Vis (MeOH): λ_max_ 306 nm (log ε = 3.09876 L cm^−1^ M^−1^). LR EIMS: *m/z* in amu (% abundance) 304, 302 (9, 31 in 1:3 ratio) [M-^•^Cl]^+^, 182, 180 (25, 8) [M-^•^CH_2_(Cl)Bn and H_2_O, A]^+^.

*(2'S)-5-Bromo-2-(2'-chloro-3'-methylpentanamido)benzoic acid* (**6g**): R*_f_*: 0.12 (EtOAc/*n*-hexane 3:7), 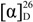 = +23.3 (*c* 0.2, MeOH); MP: 118 °C; ^1^H-NMR (500 MHz, CD_3_OD): 0.96 (3H, d, *J* = 6.5 Hz, CH_3_), 0.98 (3H, d, *J* = 6.0 Hz, H^5^^'^), 1.84–1.97 (3H, m, H^3^^'^and H^4^^'^), 4.51 (1H, dd, *J* = 9.0, 5.0 Hz, H^2^^'^), 7.69 (1H, dd, *J* = 9.0, 2.5 Hz, H^4^), 8.17 (1H, d, *J* = 2.5 Hz, H^3^), 8.53 (1H, d, *J* = 9.0, H^6^); ^13^C-NMR (125 MHz, CD_3_OD) δ (ppm): 21.59, 23.06 (CH_3_ and C^5^^'^), 25.53 (C^4^^'^), 45.49 (C^3^^'^), 60.53 (C^2^^'^), 116.75 (C^1^), 119.97 (C^5^), 123.20 (C^4^), 135.02 (C^3^), 137.87 (C^6^), 140.89 (C^2^), 169.76 (NC=O), 170.26 (OC=O); IR (KBr): ύ_max_ (cm^−1^) 3259 (O-H), 3044 (N-H), 1685 (OC=O), 1531 (NC=O); UV-Vis (MeOH): λ_max_ 310 nm (log ε = 3.23921 L cm^−1^ M^−1^); LR EIMS: *m/z* in amu (% abundance) 351, 349 and 347 (4, 12 and 11) [M]^+•^, 226, 224 (62, 43) [M-∙CH_2_(Cl)Bn and H_2_O, A]^+^, 217, 215 (99, 100) [M-∙CH_2_(Cl)Bn and CO]^+^, 199, 197 (61, 60) [A-CO]^+^.

*(3R)-8-Chloro-3-methyl-4,1-benzoxazepine-2,5-dione* (**4d**): A mixture of **6a** (1 mmol, 1 eq) and anhydrous K_2_CO_3_ (1.5 mmol, 1.5 eq) in DMF (1 mL) was heated at 80 °C for 3 h. Excess of chilled H_2_O was added, the mixture was neutralized with dilute HCl (5 mL) and extracted with EtOAc (2 × 15 mL). The combined organic layer was dried over anhydrous Na_2_SO_4_, filtered and concentrated under reduced pressure to afford the crude product. It was purified by column chromatography using 5% EtOAc in *n*-hexane as mobile phase to afford pure **4d**. R*_f_*: 0.57 (EtOAc/*n*-hexane 3:7); 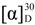 = +54.0 (*c* 0.2, MeOH); MP: 134 °C; ^1^H-NMR (400 MHz, CDCl_3_): δ (ppm) 1.61 (3H, d, *J* = 6.8 Hz, H^1^^'^), 4.79 (1H, q, *J* = 4.8 Hz, H^3^), 7.00 (1H, d, *J* = 1.6 Hz, H^9^), 7.26 (1H, dd, *J* = 8.4, 1.6 Hz, H^7^), 7.92 (1H, d, *J* = 8.4 Hz, H^6^), 7.94 (1H, s NH); IR (KBr): ύ_max_ (cm^−1^) 3262 (N-H), 1707 (a broad signal of OC=O and NC=O); UV-Vis (MeOH): λ_max_ 302 nm (log ε = 3.98631 L cm^−1^ M^−1^); LR EIMS: *m/z* in amu (% abundance) 227 and 225 (10 and 30 in 1:3) [M]^+•^, 155 and 153 (30 and 100 in 1:3 ratio) [M-(3-methyloxirane-2-one)]^+•^, 183 and 181 (3 and 10 in 1:3 ratio) [M-CO]^+•^.

## 4. Conclusions

This strategy leads towards the one-pot synthesis of novel (3*R*)-4,1-benzoxazepines-2,5-diones exploiting the chiral pool methodology. The use of (*S*)-2-bromopropanoic acid results in the formation of (3*R*)-4,1-benzoxazepines-2,5-diones with chances of racemization due to transhalogenation; on the other hand the use of (*S*)-2-chloroacids affords (*S*)-*N*-acylanthranilic acids exclusively although another base mediated step is mandatory to achieve the same product but with high *ee* [12]. Thus, the use of (*S*)-2-chloroacids for such coupling reactions is recommended to achieve high *ee* for the synthesis of (3*R*)-4,1-benzoxazepines-2,5-diones. In future, these (3*R*)-4,1-benzoxazepines-2,5-diones shall be available for various biological applications, a few of which are currently under examination.
